# A single vaccination with four-segmented rift valley fever virus prevents vertical transmission of the wild-type virus in pregnant ewes

**DOI:** 10.1038/s41541-020-00271-7

**Published:** 2021-01-08

**Authors:** Paul J. Wichgers Schreur, Judith Oymans, Jet Kant, Sandra van de Water, Anna Kollár, Yves Dehon, Pál Soós, Zoltán Pénzes, Lucien van Keulen, Jeroen Kortekaas

**Affiliations:** 1 Department of Virology, Wageningen Bioveterinary Research, Wageningen University and Research, Lelystad, The Netherlands; 2 BunyaVax B.V., Lelystad, The Netherlands; 3 Laboratory of Virology, Wageningen University and Research, Wageningen, The Netherlands; 4 Ceva Animal Health, Ceva-Phylaxia, Budapest, Hungary

**Keywords:** Biotechnology, Immunology, Microbiology, Diseases, Medical research

## Abstract

Rift Valley fever virus (RVFV) is a mosquito-transmitted bunyavirus that causes severe outbreaks among wild and domesticated ruminants, of which sheep are the most susceptible. Outbreaks are characterised by high mortality rates among new-born lambs and abortion storms, in which all pregnant ewes in a flock may abort their foetuses. In endemic areas, Rift Valley fever (RVF) can be controlled by vaccination with either inactivated or live-attenuated vaccines. Inactivated vaccines are safe for animals during all physiological stages, including pregnancy. However, optimal efficacy of these vaccines depends on multiple vaccinations and yearly re-vaccination. Live-attenuated vaccines are generally highly efficacious after a single vaccination, but currently available live-attenuated vaccines may transmit to the ovine foetus, resulting in stillbirths, congenital malformations or abortion. We have previously reported the development of a novel live-attenuated RVFV vaccine, named RVFV-4s. This vaccine virus was created by splitting the M genome segment and deleting the major virulence determinant NSs, and was shown to be safe even for the most susceptible species, including pregnant ewes. The demonstrated efficacy and safety profile suggests that RVFV-4s holds promise for veterinary and human application. The RVFV-4s vaccine for veterinary application, here referred to as vRVFV-4s, was shown to provide complete protection after a single vaccination of lambs, goats and cattle. In this work, we evaluated the efficacy of the vRVFV-4s vaccine in pregnant ewes. Anticipating on the extremely high susceptibility of pregnant ewes for RVFV, both a single vaccination and double vaccination were evaluated in two independent experiments. The combined results suggest that a single vaccination with vRVFV-4s is sufficient to protect pregnant ewes and to prevent transmission to the ovine foetus.

## Introduction

Rift Valley fever is a disease of ruminants and humans that is caused by Rift Valley fever virus (RVFV), a mosquito-borne virus of the order *Bunyavirales* (family *Phenuiviridae*, genus *Phlebovirus*). The RVFV genome is divided into three RNA genome segments of negative polarity. The large (L) segment encodes the viral RNA-dependent RNA polymerase, the medium (M) segment encodes a polyprotein precursor that is co-translationally cleaved by host proteases into the structural glycoproteins Gn and Gc, which are involved in attachment to target cells and fusion of the viral and endosomal membranes, respectively. The M segment additionally encodes a small 14-kDa protein, named NSm, which was shown to counteract apoptosis^1^, and a large glycoprotein (LGp, 78-kDa), that comprises the NSm and Gn coding regions^2^. The latter was shown to be important for dissemination of the virus in mosquitoes^3^. The S segment encodes the nucleocapsid protein and a non-structural protein named NSs. NSs efficiently counteracts several signalling pathways of the host immune system and is considered the major virulence determinant of the virus^4^.

RVFV is pathogenic to wild-ruminants and domesticated-ruminants including goats, cattle, buffalo and camelids, with sheep being the most susceptible and the most severely affected^5^. Necropsy of fatal cases may reveal widespread liver necrosis, hydrops ascites and haemorrhagic manifestations. Human infections are generally attributed to contact with contaminated animal products, predominantly blood released during the slaughtering of diseased animals, however humans may also become infected after the bite of an infected mosquito. Most infected humans develop a self-limiting febrile illness, while a small fraction develops neurological disorders or haemorrhagic fever^6^.

Apart from massive mortality among new-born lambs, abortion storms are a hallmark of RVF epizootics. A recent study on the pathogenesis of RVFV in pregnant ewes demonstrated that RVFV replicates efficiently in the ovine placenta, targeting maternal epithelial cells and foetal trophoblasts, resulting in placental demise and abortion^7^. Considering that the ovine placenta is the main source of progesterone from mid-gestation until term, destruction of the placenta is considered the primary cause of abortion. Although the risk of RVFV infection during human pregnancies is unclear, the infection has been associated with miscarriage and the virus was shown to replicate in human placental explants^7–9^.

Veterinary vaccines based on inactivated or live-attenuated virus have been marketed in several African countries. Inactivated vaccines can be applied safely during all physiological stages, including pregnancy, but require repeated dosing and yearly re-vaccination for optimal efficacy. These vaccines are therefore not ideal for emergency vaccination. Live-attenuated vaccines are either based on the Smithburn or Clone 13 strain. The Smithburn strain was derived from a mosquito isolate that was attenuated by intracerebral passage in mice^10^. Although this vaccine is efficacious after a single vaccination, its residual virulence makes it unsafe for pregnant animals^11^. Clone 13 is an alternative live-attenuated vaccine that is prescribed for use also in pregnant animals. Clone 13 is a plaque-purified clone of strain 74HB59 and found to contain a 69% deletion in the NSs gene^12,13^. This deletion was shown to render the virus avirulent for sheep lambs^14^, goats^15^, cattle^16^ and pregnant ewes^17^. Due to its high efficacy and safety profile, Clone 13 was more recently also evaluated for use in Europe according to the guidelines of the European Pharmacopeia which involves safety evaluation at an overdose. In this study, the vaccine was confirmed to be completely safe for lambs, however inoculation of an overdose in pregnant ewes was associated with stillbirths and congenital malformations^14^.

We previously reported the development of a novel live-attenuated RVF vaccine, that was constructed by splitting the M segment into two M-type segments, one encoding NSm, Gn and LGp and one encoding Gc^18^. To optimise the safety profile, the NSs gene was deleted from the S segment. The resulting four-segmented RVFV candidate vaccine was shown to be safe for pregnant ewes^19^ and young lambs^20^, even after application of an overdose, and to induce protective immunity in young sheep, goats and cattle^20^. Moreover, additional safety studies demonstrated that the vRVFV-4s vaccine does not induce viremia and is not shed or spread to the environment^20^. In this study, the efficacy of two independent batches of vRVFV-4s (referring to the RVFV-4s vaccine for veterinary application) were evaluated in pregnant ewes. Considering the very high susceptibility of pregnant ewes, both a single vaccination and a double vaccination with different doses were evaluated. The results demonstrate that both a single vaccination and a double vaccination at a lower dose with vRVFV-4s prevents vertical transmission of a highly virulent challenge strain.

## Results

### Efficacy of vRVFV-4s in pregnant ewes (Experiment 1)

**Fig. 1:**
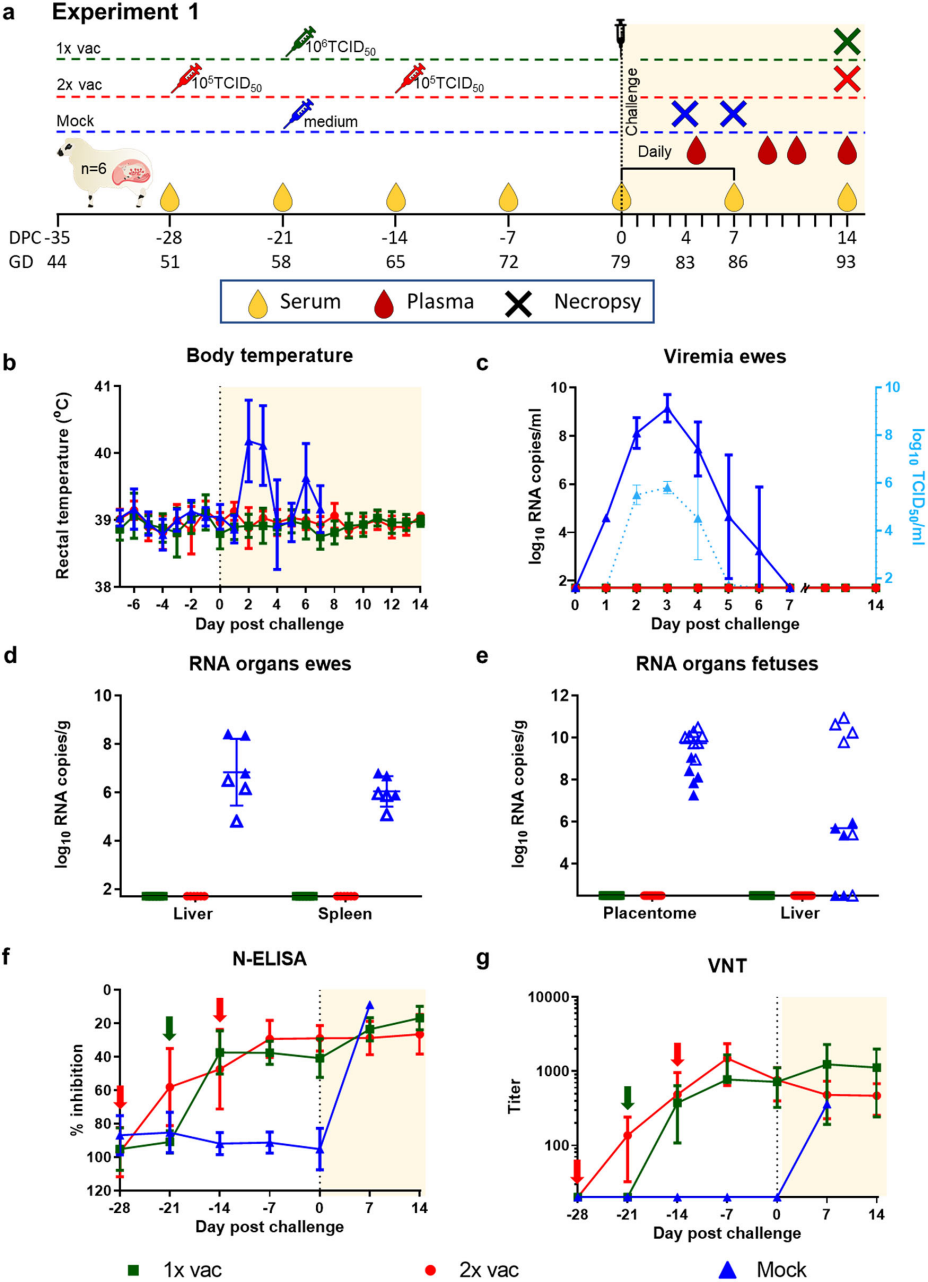
Primary outcome parameters of vaccination challenge Experiment 1. **a** Graphical representation of the experiment. Pregnant ewes were vaccinated either once or twice at the time points indicated and challenged two weeks (double vaccination) or three weeks (single vaccination) after vaccination with virulent RVFV strain 35/74. Vaccinated/challenged ewes were euthanized 14 days post challenge. Ewes in the mock group were euthanized on day 4 post challenge (*n* = 3) or at imminent abortion (*n* = 3). **b** Rectal temperatures in °C. **c** Monitoring of viral RNA by RT-qPCR (detection limit 1.3 log_10_ RNA copies/ml). Samples with an RNA copy number of >5 log_10_/ml were assayed for infectious virus by virus isolation on BHK cells (detection limit of 1.55 log_10_ TCID_50_/ml). **d** Detection of viral RNA in liver and spleen samples of the ewes (detection limit 2.3 log_10_ RNA copies/gram). Closed symbols represent samples collected on DPC 4, open symbols represent samples collected on DPC 7. **e** Detection of viral RNA in placentomes and foetal livers (detection limit 2.3 log_10_ RNA copies/gram). Of each placenta, one placentome was tested. **f** Detection of anti-N antibodies by competition ELISA in weekly obtained sera. Competition is expressed as percentage inhibition ratio of the optical densities (OD) of the sample and the OD of the negative control (% S/N). All values below 40% are considered positive, between 40% and 50% are considered doubtful and above 50% are considered negative. **g** Detection of neutralising antibody responses by VNT^29^. Moments of vaccination are indicated in panels **f** and **g** by arrows. Measurements were taken from distinct samples. Error bars represent s.d.

**Fig. 2:**
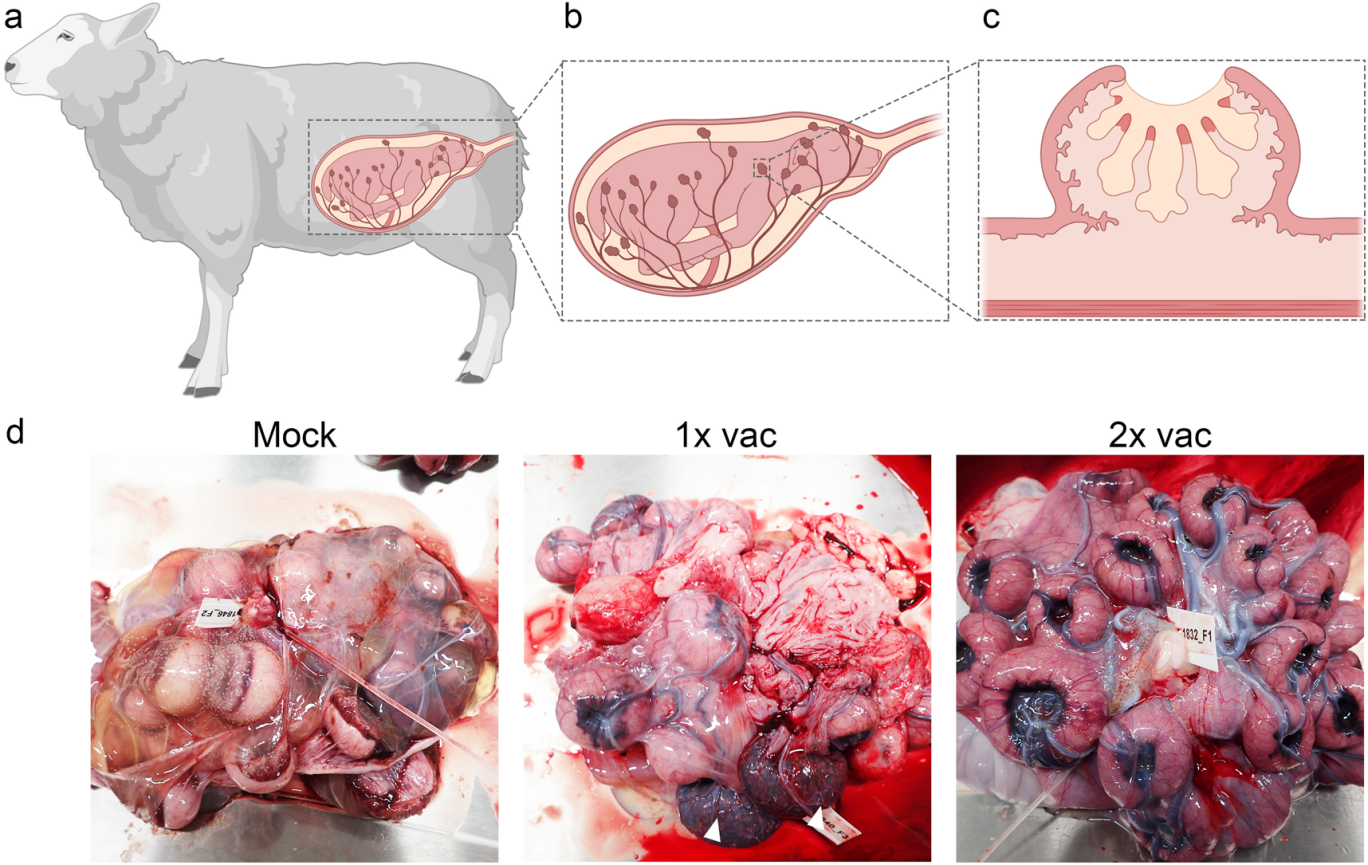
Macroscopic assessment of placentas from mock-vaccinated ewes and ewes vaccinated with vRVFV-4s followed by challenged with wild-type RVFV. **a** Cartoon of a pregnant ewe including a magnification of the ovine placenta (**b**) with placentomes and a magnification of a single placentome (**c**), revealing the foetal (beige) and maternal (pink) villi and haemophagous zone (red). **d** Representative pictures of placentas from a mock-vaccinated challenged ewe (left panel; ewe #1846, foetus #F2), a challenged ewe that was vaccinated once (middle panel; ewe #1840, foetus #F3), and a challenged ewe that was vaccinated twice (right panel; ewe #1832, foetus #F1). Note the two type D placentomes in the placenta of the 1× vaccinated ewe (white arrowheads in middle panel, see also Supplementary Fig. 2). The cartoons in **a**, **b** and **c** were created using BioRender.com.

The first experiment with pregnant ewes was performed with an investigational batch of vRVFV-4s^18^. Eighteen pregnant ewes (synchronised pregnancy) were divided over three groups of 6 animals. Group “1× vac” (#1835–1840) was vaccinated with a dose of 10^6^ TCID_50_ via intramuscular (IM) route on gestation day (GD) 58. On the same day, the Mock group (#1841–1846) was inoculated with culture medium. Group “2× vac” (#1829–1834) was vaccinated twice, on days 51 and 65 of gestation, with a dose of 10^5^ TCID_50_. Three weeks post single vaccination and two weeks post double vaccination, all ewes were challenged via intravenous (IV) route with 10^5^ TCID_50_ of RVFV strain 35/74, previously rescued in BSR-T7 cells and amplified in BHK-21 cells^21^. Three of the mock-vaccinated ewes (#1841–1843) were euthanized on day 4 post challenge (DPC), whereas the remaining three ewes were euthanized at imminent abortion. The outline of the study is presented in Fig. 1a. No untoward effects or other clinical signs were observed following vaccination. All mock-vaccinated ewes manifested with elevated rectal temperatures (Fig. 1b) and viremia (Fig. 1c) following challenge infection. Necropsy of mock-vaccinated ewes on DPC 4 revealed multifocal necrotising hepatitis, although placentas revealed no macroscopic abnormalities and all foetuses were still alive. In the morning of DPC 7, one mock-vaccinated ewe (#1844) had expelled 2 foetuses and a second ewe (#1845) was in the process of aborting, with two foetuses already expelled and one foetus still in the uterus. Necropsy of the third ewe (#1846) revealed three foetuses that were still inside the uterus and placentomes showing extensive haemorrhages, and varying degrees of cotyledonal detachment (Fig. 2). One of these foetuses was alive, whereas the remaining two foetuses were found dead (Supplementary Fig. 1). Analysis of liver and spleen samples revealed very high viral RNA levels in the organs of ewes necropsied on 4 DPC and lower levels in ewes necropsied at 7 DPC (Fig. 1d). High levels of viral RNA were detected in all placentomes and most of the foetal livers (Fig. 1e). Details about virological and (histo)pathological findings in ewes and foetuses were reported previously^7^ as the mock-vaccinated group was also part of an experiment in which the pathology of RVFV for pregnant ewes was assessed.

Ewes that had received a single vaccination and were challenged three weeks later did not present with increased rectal temperatures (Fig. 1b) and no viremia (Fig. 1c) was detected. At necropsy on 14 DPC, the ewes were found to carry a total of 18 live, apparently healthy foetuses with crown-rump lengths of about 25 cm (as expected for their gestation stage). It was interesting to find that in two ewes (#1838 and #1840) some placentas revealed not only concave, type A/B placentomes but also convex, type C and D placentomes (Fig. 2d, Supplementary Fig. 2, and Supplementary Tables 1a, b).

Ewes that had received a double vaccination and were challenged two weeks later also did not develop signs of disease. Also in these ewes, no rises in rectal temperatures (Fig. 1b) or viremia (Fig. 1c) were detected. At necropsy (15 DPC), these ewes were found to carry a total of 10 live, and apparently healthy foetuses with crown-rump lengths of about 26 cm (also as expected for their gestation stage, Supplementary Fig. 1). No viral RNA was detected in maternal or foetal organs of any of the vaccinated animals (Fig. 1d, e).

All vaccinated ewes developed anti-nucleocapsid (N) antibodies, as determined by ELISA (Fig. 1f), as well as virus-neutralising antibodies as determined by VNT (Fig. 1g). No significant differences in the antibody levels at the time of challenge were observed between the vaccinated groups.

### Efficacy of a MSV+5 of vRVFV-4s in pregnant ewes (Experiment 2)

After the promising results obtained from the first study with the investigational batch of vRVFV-4s, a master seed virus (MSV) was developed by passage of the virus in BSR-T7 cells^20^, wich was subsequently passaged 5 times in the same cells in this work. This MSV+5 batch was subsequently evaluated in a second study with pregnant ewes in full compliance with the European Pharmacopeia (EP) monograph 5.2.7 (Evaluation of efficacy of veterinary vaccines and immunosera). Moreover, even lower doses for the 1× vac and 2× vac groups were assessed, and necropsies were scheduled at 3 weeks, instead of 2 weeks after challenge.

**Fig. 3:**
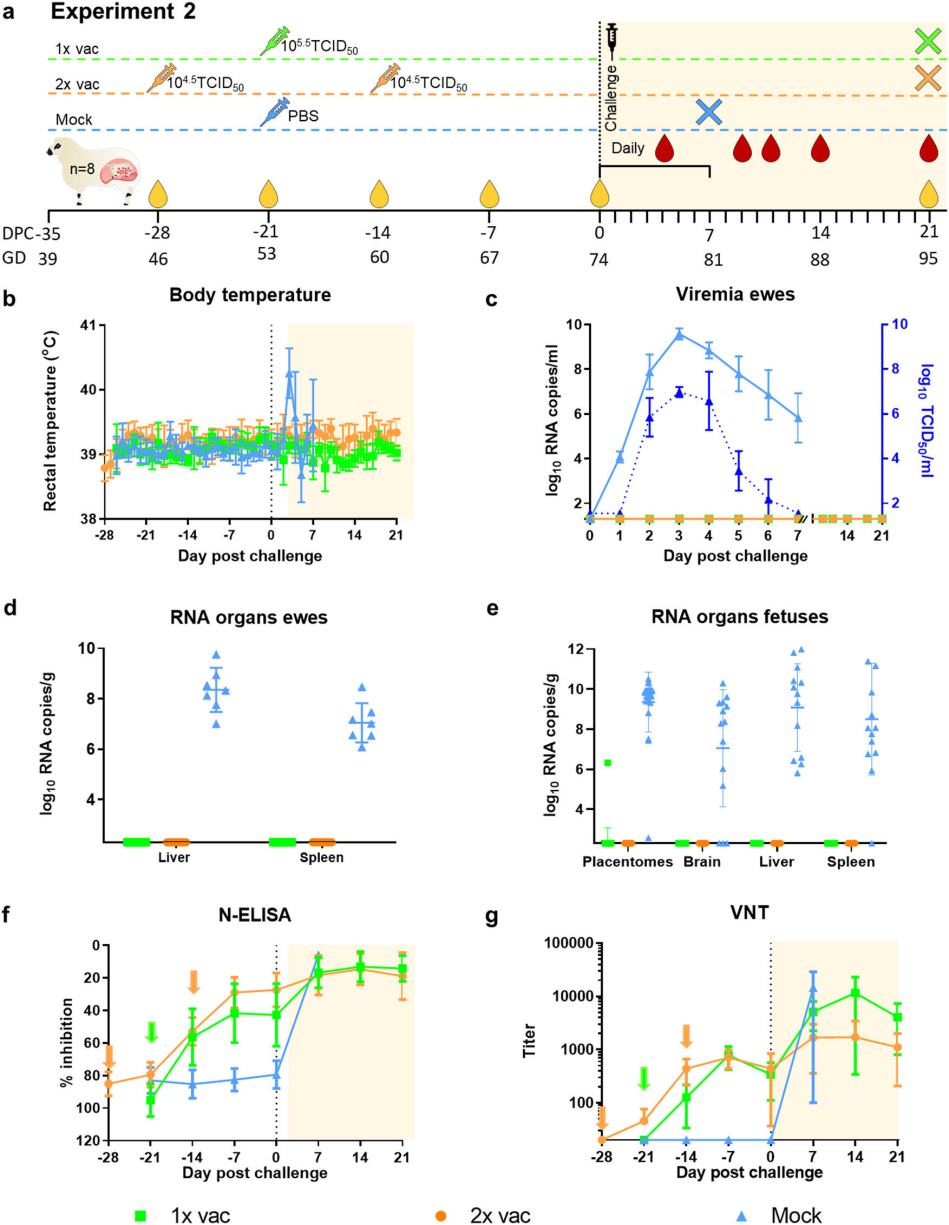
Primary outcome parameters of vaccination challenge Experiment 2. **a** Experimental design. Pregnant ewes were vaccinated either once or twice at the time points indicated and challenged two weeks (double vaccination) or three weeks (single vaccination) after vaccination with highly virulent RVFV strain 35/74. Vaccinated/challenged ewes were euthanized 21 days post challenge. One ewe in the mock group succumbed on day 5, whereas the other ewes were euthanized at 7 DPC. **b** Rectal temperatures in °C. **c** Monitoring of viral RNA by RT-qPCR (detection limit 1.3 log_10_ RNA copies/ml). Samples with an RNA copy number of >10^5^/ml were assayed for viremia by virus isolation on BHK cells (detection limit of 1.55 log_10_ TCID_50_/ml). **d** Detection of viral RNA in liver and spleen samples of the ewes (detection limit 2.3 log_10_ RNA copies/gram). **e** Detection of viral RNA in placentomes, brains, liver and spleens of foetuses (detection limit 2.3 log_10_ RNA copies/gram). Of each placenta, two placentomes were tested. **f** Detection of anti-N antibodies by competition ELISA in weekly obtained sera. Competition is expressed as percentage inhibition ratio of the optical densities (OD) of the sample and the OD of the negative control (% S/N). All values below 40% are considered positive, between 40 and 50% are considered doubtful and above 50% are considered negative. **g** Detection of neutralising antibody responses by VNT. Moments of vaccination are indicated in panels **f** and **g** by arrows. Measurements were taken from distinct samples. Error bars represent s.d.

Twenty four ewes were divided over 3 groups of 8 animals. The first group “1× vac” (#207–214) was vaccinated with a dose of 10^5.5^ TCID_50_ via IM route on GD 53. The Mock group (#223–230) was inoculated with PBS on the same day. Group “2× vac” (#199–206) was vaccinated twice with a dose of 10^4.5^ TCID_50_ on GDs 46 and 60. Three weeks post single vaccination and two weeks post double vaccination, all ewes were challenged, via IV route, with 10^5^ TCID_50_ of RVFV strain 35/74. The outline of the study is presented in Fig. 3a. In line with experiment 1, no clinical signs or other untoward events were noted after vaccination. One ewe from the control group (#226) developed laryngeal chondritis prior to challenge and had to be euthanized and removed from the experiment. After challenge, 6 of 7 mock-vaccinated ewes developed increased rectal temperatures with onset on day 3 (Fig. 3b), associated with high viremia levels as determined by RT-qPCR and virus isolation (Fig. 3c). One ewe acutely died on DPC 5 (#228). Necropsy of this ewe revealed a necrotic liver, haemorrhagic placentas and two dead foetuses. One ewe aborted one foetus on DPC 7. To prevent unnecessary animal discomfort, all remaining ewes were necropsied on this day as well. In total, the mock-vaccinated ewes were found to carry 14 foetuses, which had all succumbed to the infection.

Analysis of samples from mock-vaccinated ewes and their foetuses revealed very high viral RNA levels in maternal liver and spleen samples (Fig. 3d), and high levels of viral RNA in all placentomes, foetal brains, livers, and spleens (Fig. 3e). Of note, the mock-vaccinated group was shared with another experiment in which the Chimpanzee Adenovirus Oxford-based RVF (ChAdOx1-RVF) vaccine was evaluated^22^.

In contrast to the mock-vaccinated ewes, all vRVFV-4s-vaccinated ewes did not show any clinical symptoms that could be attributed to the RVFV challenge. Ewes that were vaccinated once with 10^5.5^ TCID_50_ were found to carry a total of 16 foetuses, whereas ewes vaccinated twice with 10^4.5^ TCID_50_ were found to carry a total of 18 foetuses. All 34 foetuses appeared healthy and were of the expected crown-rump size (between 26 and 28 cm). All placentas of vaccinated ewes appeared healthy. Nevertheless, in two ewes (#207 and #214), vaccinated once with 10^5.5^ TCID_50_, the placentas of 4 foetuses revealed not only concave, type A/B placentomes but also convex, type D placentomes (Supplementary Fig. 2, Supplementary Tables 2a, b). Similar as in experiment 1, this was observed only in 1× vaccinated ewes, and not in 2× vaccinated ewes. Again in line with experiment 1, no viral RNA was observed in the organs (livers and spleens) of vaccinated ewes except for 1 out of 32 tested placentomes in the 1× vac group in which a low level of viral RNA was detected by RT-qPCR. Importantly, no viral RNA was detected in any tested foetal tissue sample.

Similar as in the first experiment, all vaccinated ewes seroconverted, as determined by ELISA (Fig. 3f) and VNT (Fig. 3g), and again no significant differences at the time of challenge were observed between the vaccinated groups.

### Macroscopic assessment and histopathology of placental tissues

**Fig. 4:**
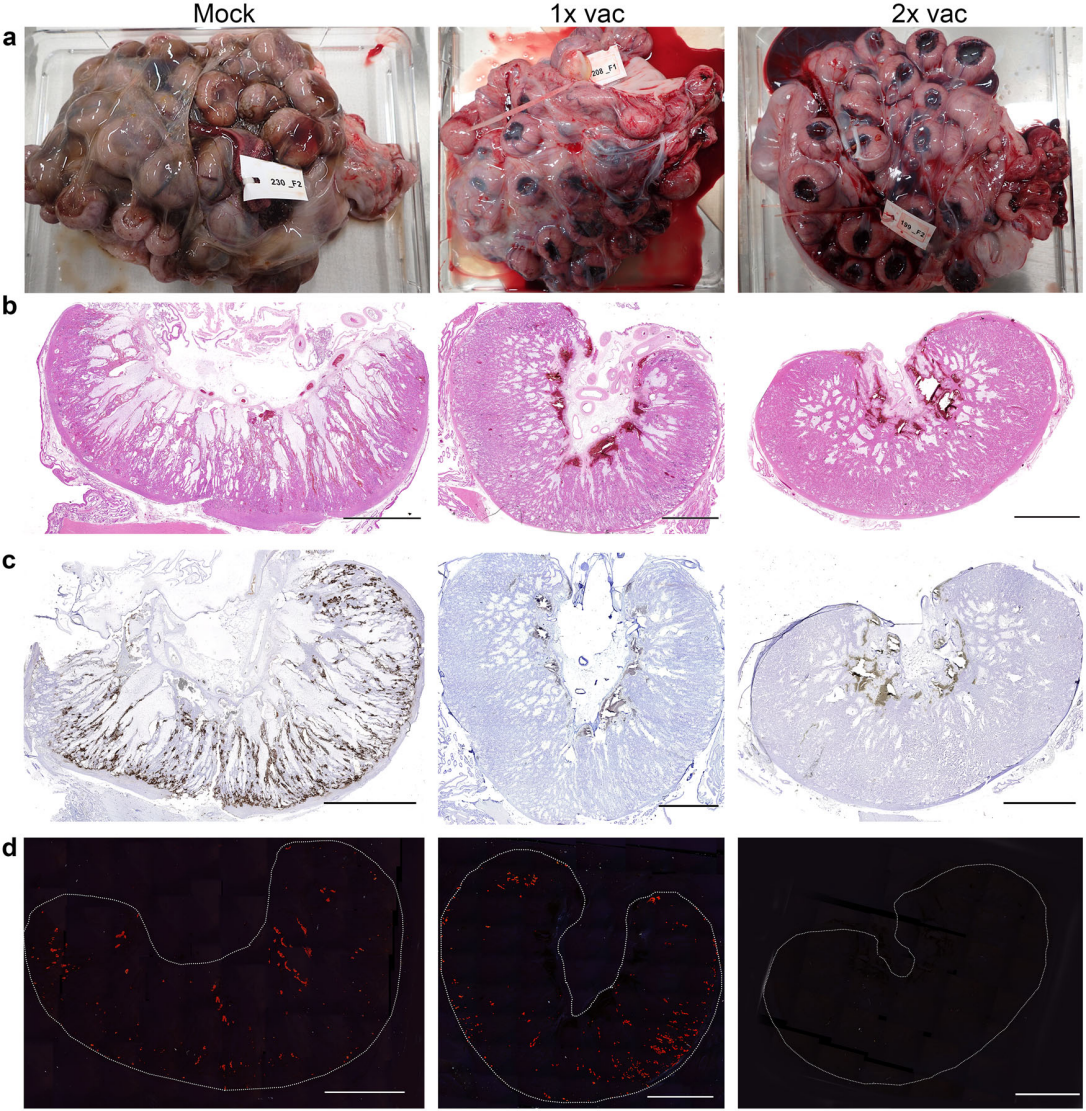
Macroscopic assessment of placentas and histopathology of placentomes from mock-vaccinated ewes and ewes vaccinated with vRVFV-4s MSV+5 and challenged with RVFV strain 35/74. **a** Placenta from a mock-vaccinated, challenged ewe (ewe #230, foetus #F2), euthanized at 7 days post challenge (left panel). Placenta from a 1× vaccinated ewe (ewe #208, foetus #F1), euthanized at 24 days post challenge (centre panel). Placenta from a 2× vaccinated ewe (ewe #199, foetus #F2) euthanized at 21 days post challenge (right panel). **b** H&E staining of representative placentome sections, **c** IHC staining of placentome sections with RVFV-specific mAb 4-D4, **d** Alizarin Red staining of placentome sections visualised using polarised light. Bars represent 5 mm.

Similar as in Experiment 1, placentas from mock-vaccinated ewes were severely affected showing haemorrhages, necrosis and cotyledonal detachment, whereas placentas from vaccinated ewes appeared healthy (Fig. 4a). However, the observed morphological changes of some placentomes and the detection of viral RNA in one placentome in challenged ewes that had received a single vaccination prompted a more detailed analysis of the placentas by (immuno)histochemistry. H&E staining of placentomes from mock-vaccinated and subsequently challenged ewes revealed extensive haemorrhages and necrosis of maternal epithelium (Fig. 4b, left panel) associated with RVFV antigen both in maternal and foetal epithelial cells (Fig. 4c, left panel). Dystrophic calcification of necrotic maternal epithelial cells was visualised by Alizarin Red staining (Fig. 4d, left panel).

Placentomes from vaccinated ewes appeared unaltered by H&E staining (Fig. 4b, centre and right panels), and absence of viral antigen, in line with the PCR results, was confirmed by IHC (Fig. 4c, middle and right panels), although foci with calcium deposits in the maternal epithelium were detected in some ewes that had received a single vaccination (Fig. 4d, middle panel, see also Supplementary Table 2a, b). This suggests that in a few ewes that had received a single vaccination some local virus infection/replication had taken place following challenge. However, the virus was apparently cleared rapidly as low-level viral RNA was detected in only 1 out of 32 placentomes tested at three weeks post challenge (1× vac group, Fig. 3e). Importantly, no viral antigen was detected in foetal tissues, suggesting that a single vaccination was sufficient to prevent vertical transmission.

## Discussion

We previously reported that a single vaccination with vRVFV-4s protects young sheep, goats and cattle from virulent RVFV challenge^20^. Here, we show that a single vaccination protects pregnant ewes and their foetuses against wild-type RVFV challenge in two independent experiments. Anticipating on the high susceptibility of pregnant ewes for wild-type RVFV, we evaluated both a single vaccination and a double vaccination at two different doses. Whereas neutralising antibody levels did not differ significantly among the groups, challenge infection resulted in a higher increase in antibody responses in ewes that had received a single vaccination with a dose of 10^5.5^ TCID_50_, suggesting that some challenge virus may have replicated in the placentas of these ewes. This is supported by the detection of a low level of viral RNA in one placentome at necropsy.

Sheep placentomes can be classified into four types: A, B, C, and D, with type A and B being predominant under normal conditions (Supplementary Fig. 2)^23^. Type C and D placentomes represent eversions of the haemophagous zone, which are associated with physical adaptations to stressors, such as hypoxia or malnutrition, considered to facilitate exchange of oxygen and nutrients^24^. However, it is important to note that eversion of the haemophagous zone may also occur at late pregnancy and is not associated with pathological changes^25^. In ewes that received a double vaccination, only type A and B placentomes were observed, whereas in some challenged ewes that had received a single vaccination, type C and D placentomes were found. These placentome eversions may have resulted from localised placental lesions that also explain the observed calcifications^26^. Similar calcifications were previously observed in ewes that were vaccinated with the ChAdOx1-RVF vaccine candidate following challenge^22^. If indeed limited and transient challenge virus replication occurred in maternal epithelium of the placenta, it is quite remarkable that maternal immunity prevented transmission of the challenge virus to foetal trophoblasts, directly lining the maternal epithelium and previously shown to be highly susceptible to RVFV^7^.

In conclusion, our results suggest that a single vaccination of pregnant ewes provides complete protection from vertical transmission and abortion. Considering that placentas and foetuses expelled from RVFV infected ewes pose a serious risk for farmers and veterinarians handling these materials, vaccination with vRVFV-4s could additionally prevent human morbidity and mortality.

## Methods

### Cells and viruses

Culture media and supplements were obtained from Gibco unless indicated otherwise. Baby Hamster Kidney (BHK-21) and BSR-T7 cells were maintained in Glasgow minimum essential medium (GMEM) supplemented with 4% tryptose phosphate broth, 1% minimum essential medium nonessential amino acids (MEM NEAA), 1% antibiotic/antimycotic (a/a) and 5% foetal bovine serum (FBS), at 37 °C with 5% CO_2_.

The investigational batch of vRVFV-4s (previously referred as RVFV-LMMS_delNSs_) was produced following low MOI infection (MOI 0.005) of BSR-T7 cells with rescue supernatant obtained by reverse genetics^18^. The MSV stock was prepared by infecting BSR-T7 cells with plaque-purified seed virus, in complete medium (GMEM supplemented with 5% FCS (SAFC), 4% TPB, 0.001% Gentamycin and 1% NeAA) at a multiplicity of infection (MOI) of 0.002. BSR-T7 cells were used, as amplification of the vaccine virus in these cells yields high titres. Both the investigational batch and the MSV stock were harvested at 3 days post infection (DPI). To produce the MSV+5 stock the virus was passaged 5 times in BSR-T7 cells using an MOI of 0.002 with harvesting at 3 DPI.

At 3 DPI, immunoperoxidase monolayer assays were performed and titres calculated using the Spearman-Kärber algorithm. Recombinant RVFV strain 35/74, was propagated in BHK-21 cells, which were grown in CO_2_-independent medium (Gibco), supplemented with 5% FCS (Gibco), 1% l-glutamine (Gibco) and 1% antibiotic/antimycotic (anti/anti, Gibco) and used as challenge virus^21^.

### Experimental design pregnant ewe trials

Two experiments were performed to determine the efficacy of the vRVFV-4s vaccine in pregnant ewes. For both experiments, ewes were treated with progesterone sponges to synchronise pregnancy after which the ewes were naturally mated. At 6–7 weeks post conception, pregnancy was confirmed via ultrasound and the general health of the ewes was assessed by a veterinarian.

### Efficacy of an investigational batch of vRVFV-4s in pregnant ewes (Experiment 1)

Eighteen pregnant ewes were transported to the BSL-3 facility of WBVR, where they were allowed to acclimatise for 7 days. Serum was collected weekly from that moment onwards (Fig. 1a). At day 51 and 65 of gestation, the group that was to be vaccinated twice was vaccinated with 10^5^ TCID_50_ of the vRVFV-4s vaccine. At GD 58, the 1× vac group was vaccinated with 10^6^ TCID_50_ and the Mock group with culture medium. Three weeks later, the ewes were challenged by IV inoculation of 10^5^ TCID_50_ of recombinant RVFV^27^. Ewes were observed twice per day for general health and signs of abortion and rectal temperatures were recorded daily. EDTA blood samples were collected daily for the first seven days and at specific timepoints thereafter (9, 11 and 14 days DPC). At 14 DPC, or when a humane endpoint (HEP) was reached, the ewes and their foetuses were euthanized by IV administration of 50 mg/kg sodium pentobarbital (Euthasol, ASTfarma) and subsequent exsanguination. From the ewes samples were taken from the liver and spleen. From the foetus, samples were taken from the liver and from one placentome. Samples were either placed on dry ice and stored at −80 °C for virus isolation and RNA extraction or samples were fixed in 10% phosphate-buffered formalin for at least 48 h before routine processing into paraffin blocks.

### Efficacy of MSV+5 in pregnant ewes (Experiment 2)

To study efficacy of the MSV+5 in pregnant ewes, twenty-four ewes were transported to WBVR at day 39 of pregnancy, after which the animals were allowed to acclimatise for 1 week. Ewes that received a single vaccination (1× vac), were inoculated via IM route with a dose of 10^5.5^ TCID_50_ on GD 53. On the same day, the Mock group was inoculated with PBS. Ewes that received two vaccinations, were inoculated with a dose of 10^4.5^ TCID_50_ on GD 46 and 60 (2× vac; Fig. 3a). On GD 74, all groups were challenged via IV route with 10^5^ TCID_50_ of recombinant RVFV. Rectal temperatures were measured daily and each day the ewes were observed twice for general health and signs of abortion. Serum samples were taken weekly from GD 46 and plasma was collected daily after challenge for the first week and at intervals thereafter (9, 11, 14 and 21 DPC). All ewes were euthanized at 21 DPC, or when a HEP was reached, by IV administration of 50 mg/kg sodium pentobarbital (Euthasol, ASTfarma) and subsequent exsanguination. Samples were collected from the liver and spleen from the ewes. Two placentomes were collected from each placenta and foetal samples were taken from the brain, liver and spleen. Samples were either placed on dry-ice and stored at −80 °C for virus isolation or used for RNA extraction, or samples were fixed in 10% phosphate-buffered formalin for at least 48 h before routine processing into paraffin blocks. Experimental details are noted in Table 1 according to the ARRIVE guidelines^28^.

**Table 1:** Experimental details according to the ARRIVE guidelines.

	Investigational batch	MSV+5
Animals		
Species	Sheep	
Breed and/or strain	Texel cross breed	
Source	Conventional Dutch sheep farm	
Sex	Female	
Developmental stage (age)	Between 1.5 and 5 years	
Health and physiological status	Healthy and pregnant	
Weight	60 kg	
Identification	By ear tag and by non-irritating coloured spray on the back of the animal.	
Housing and Husbandry		
Type of facility	BSL-3 facility	
Type of housing	Stables of 18 m^2^	
Bedding material	Wood shavings	
Number of animals per stable	6	
Light/dark cycle	12/12	
Temperature	21 °C	23 °C
Quality of water	Tap water, quality checked daily	
Type of food	Hay, grass pellets and sheep grain	
Access to water and food	Water ad libitum, Food once per day	
Acclimatisation time	7 days	
Experimental procedure		
Number experimental groups	3	
Number of animals per group	6	8
Statistical support	In the unprotected control group, all ewes were expected to abort or to carry infected foetuses. To show protection of at least 80% in the vaccinated groups (α = 5% and β = 90%) Using the Fisher’s exact test, one-sided testing, with the software G*Power 3.0.10., we calculated that we needed at least 6 animals per group.	
Randomisation procedure	Animals were sorted by age and subsequently divided over experimental groups	
Experimental unit	Group	
Route of administration	Vaccination: IM injection right *gluteus maximus* muscle Challenge: IV injection in jugular vein	
Anaesthesia and analgesia	Not applicable	
Method of euthanasia	IV injection with sodium pentobarbital	
Humane endpoints	–The animal is recumbent and does not rise even after stimulation –The animal is unable to drink –The animal is lethargic (listless, apathic, non-responsive to stimuli) –Signs of abortion	
Observations	The animals were checked for clinical signs once per day, with an intensification of twice per day after challenge	

### Detection of viral RNA

Organ samples were homogenised by adding 0.3–1 g of tissue to an IKA Ultra Turrax Tube DT-20 containing 7 ml CO_2_-Independent Medium (CIM) supplemented with 1% a/a. The suspensions were transferred to 15 ml Falcon tubes and cell debris was removed by centrifugation for 15 min at 4952 × *g*.

Organ suspensions or plasma samples (200 µl) obtained in Experiment 1 were added to 50 μl Proteinase K (5 μg/ml, Sigma). Next, 200 μl AL buffer (Qiagen), supplemented with 2 μl polyadenylic acid A (5 mg/ml, Sigma), after which the samples were thoroughly mixed and incubated at 56 °C for 15 min. Subsequently, 250 μl 99% ethanol was added and RNA was isolated using the Qiagen RNeasy kit according to the manufacturer’s protocol.

Organ suspensions or plasma samples (500 µl) obtained in Experiment 2 were added to 2.5 ml NucliSENS easyMAG Lysis Buffer (Biomérieux, Marcy-l’Étoile, France), after which RNA was extracted using the NucliSENS easyMAG (Biomérieux) according to manufacturer’s protocol.

Five μl of the RNA was used in a RT-qPCR using the The LightCycler RNA Amplification Kit HybProbe (Roche, Almere, the Netherlands). Primers and probes were purchased from IDT. Forward primer: 5′-AAAGGAACAATGGACTCTGGTCA-3′, reverse primer: 5′-CACTTCTTACTACCATGTCCTCCAAT-3′; Probe: 5′-6FAM-AAAGCTTTGATATCTCTCAGTGCCCCAA-TMR-3′. Cycling conditions were as follows: 45 °C for 30 min, 95 °C for 5 min, 45 cycles of 5 s at 95 °C and 35 s at 57 °C, followed by cooling down to 30 °C.

### Virus isolation

Virus isolations were performed on RT-qPCR positive samples with a threshold above 10^5^ RNA copies/ml as this has been previously shown to be a cut-off point below which no live virus can be detected^19^. Virus isolations of plasma were performed by serial dilution in complete CO_2_-indepentent medium (CIM; supplemented with 5% FBS and 1% a/a) supplemented with 3.5 IU/ml heparin, and virus isolation of organ suspensions were serially diluted in complete CIM. Subsequently, the virus dilutions were incubated with BHK-21 cells. After 1.5 h incubation at RT, the inocula were replaced by fresh medium and after 5 days of culturing the cells at 37 °C and 5% CO_2_ cytopathic effects were scored.

### Virus neutralisation test and ELISA

Serum RVFV neutralising antibodies were measured using a virus neutralisation test (VNT)^29^. Briefly, serial dilutions (50 μl) of heat-inactivated sera (2 h, 56 °C) were incubated with 50 μl of RVFV-4s_eGFP_ (10^3.6^ TCID_50_/ml) for 2 h at room temperature. Subsequently, 20,000 BHK-21 cells (in 50 μl) were added to each well. Plates were incubated for 2 days at 37 °C and 5% CO_2_ and scored using an EVOS-FL microscope (Life Technologies). VNT_50_ titres were calculated using the Spearman–Kärber algorithm.

Presence of RVFV nucleoprotein-specific antibodies in sera was determined using the ID Screen® Rift Valley Fever Competition ELISA (ID-Vet, Montpellier, France).

### Histology and immunohistochemistry

Paraffin-embedded tissues were cut into 4 μm sections, collected on silane-coated glass slides and dried for at least 48 h in a 37 °C incubator. After deparaffinization and rehydration in graded alcohols, sections were stained routinely with haematoxylin and eosin (H&E) or immunostained for RVFV antigen. For immunostaining, endogenous peroxidase was blocked for 30 min in methanol/H_2_O_2_ followed by antigen retrieval through 15 min autoclaving at 121 °C in pH 6 citrate buffer (Antigen unmasking solution, Vector Laboratories). As RVFV Gn-specific primary antibody, monoclonal antibody 4-D4 was used (3 μg/ml). Specificity of the immunostaining was confirmed with 2 other mAbs directed against different proteins of RVFV. Mouse Envision peroxidase (K4007, Dako, Denmark) was used as secondary antibody and diaminobenzidine (DAB; Dako, Denmark) as the substrate, according to the manufacturer’s instructions. Hematoxylin was used to counterstain the slides. Calcium deposits were stained with Alizarin Red (Merck, Darmstadt, Germany).

### Ethics statement

Animal trials were conducted in accordance with European regulations (EU directive 2010/63/EU) and the Dutch Law on Animal Experiments (Wod, ID number BWBR0003081). Permissions were granted by the Dutch Central Authority for Scientific Procedures on Animals (Permit Number: AVD401002017816). All procedures were approved by the Animal Ethics Committees of Wageningen Research. The following HEPs were applied: (1) the animal is recumbent and does not rise even after stimulation, (2) the animal is unable to drink, (3) the animal is lethargic (listless, apathic, non-responsive to stimuli), (4) Signs of abortion.

### Reporting summary

Further information on research design is available in the Nature Research Reporting Summary linked to this article.

## Supplementary information

Supplemental Material

Reporting Summary

## Data Availability

All data necessary to interpret, replicate and build upon the methods or findings reported in the article are provided in this article.
