# Supplementary material for: A single vaccination with four-segmented rift valley fever virus prevents vertical transmission of the wild-type virus in pregnant ewes
Source: NPJ Vaccines. 2021 Jan 8;6:8. doi: 10.1038/s41541-020-00271-7 (PMC7794363; doi:10.1038/s41541-020-00271-7)
Supplement: Supplementary file 1 — Supplemental Material [file 41541_2020_271_MOESM1_ESM.pdf]

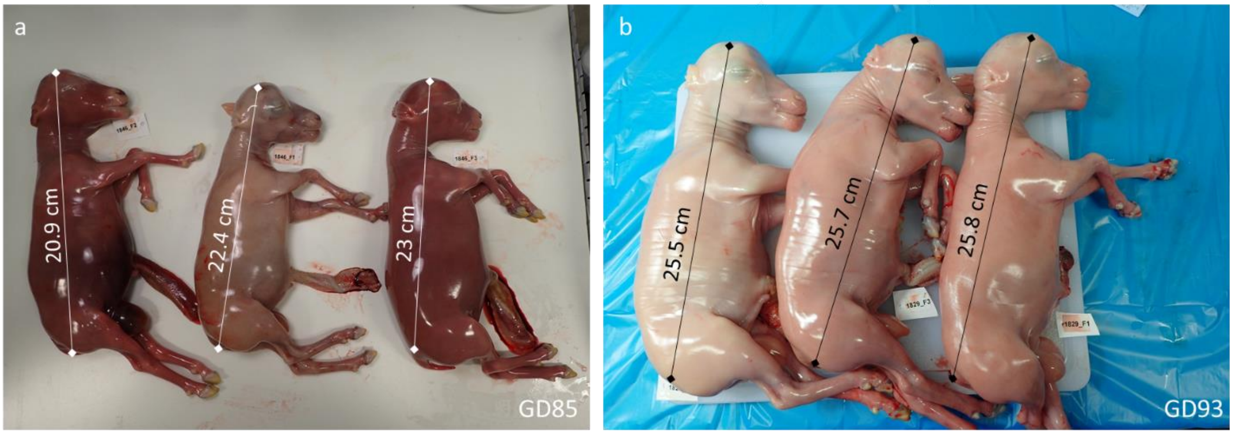

Supplementary Fig. 1. Representative pictures of foetuses from a mock-vaccinated ewe and a vaccinated ewe. **a** Foetuses collected at imminent abortion from mock-vaccinated ewe #1846 on GD85 (Experiment 1). The central foetus was alive at necropsy, whereas the two siblings were found dead. **b** Foetuses collected from ewe #1829 on GD93 (Experiment 1, 2x vac group). All thee foetuses were alive at necropsy.

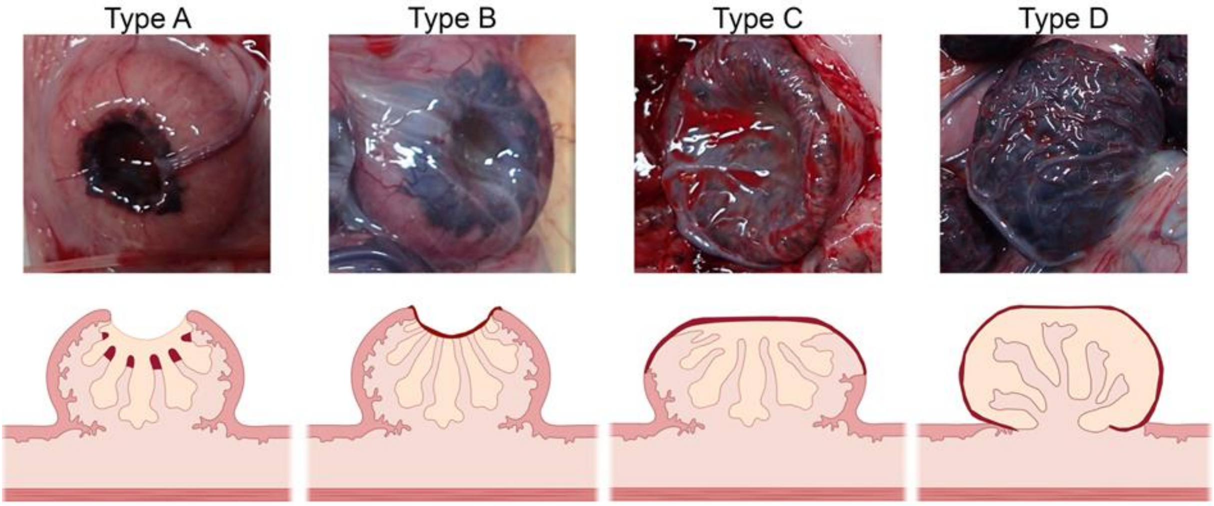

Supplementary Fig. 2. Morphology of type A, B, C and D placentomes. The haemophagous zone, indicated in red in the cartoons, is an area where maternal blood extravasates between the maternal crypts (pink) and fetal villi (beige). In type A placentomes, the haemophagous zone is for the most part positioned on the inside of the placentome. In type B placentomes, the haemophagous zone is partially everted, revealing a larger external surface area. The placentome further transits to type D placentomes via the intermediate, and characteristically flat, type C placentome. Cartoons were created using BioRender.com.

Supplementary Table 1a: Experiment 1, Group 1x vac

Ewe number #	Foetus number #	Type placentomes	Calcifications +/-
1835	F1	A	+
	F2	A	+
1836	F1	A, B	-
	F2	A, B	-
	F3	A, B	-
	F4	A, B	-
1837	F1	B	-
	F2	A, B	-
1838	F1	B, D	-
	F2	B, C, D	+
	F3	B	-
1839	F1	A, B	-
	F2	A, B	-
	F3	A, B	-
1840	F1	A	-
	F2	B, D	-
	F3	A, B, D	-
	F4	A, B, D	-

Supplementary Table 1b: Experiment 1, Group 2x vac

Ewe number #[a]	Foetus number #	Type placentomes	Calcifications +/-
1829	F1	A,B	-
	F2	A	-
	F3	A	-
1830	F1	A	-
	F2	A	-
1831	F1	A	-
1832	F1	A	-
1833	F1	A	-
1834	F1	A	-
	F2	A	-

Supplementary Table 2a: Experiment 2, Group 1x vac.

Ewe number #	Foetus number #	Type placentomes	Calcifications +/-
207	F1	A, B, D	-
	F2	A, B, D	-
	F3	D	-
208	F1	A	+
	F2	A	+
209	F1	A, B	-
	F2	A, B	-
210	F1	A, B	-
	F2	A, B	-
211	F1	B	+
	F2	B	-
212	F1	A	-
213	F1	A, B	-
	F2	A, B	-
214	F1	A, B, D	-
	F2	B	-

Supplementary Table 2b: Experiment 2, Group 2x vac.

Ewe number #	Foetus number #	Type placentomes	Calcifications +/-
199	F1	A	-
	F2	A	-
200	F1	A, B	-
201	F1	A	-
	F2	A	-
	F3	A	-
202	F1	A	-
	F2	A	-
203	F1	A, B	-
	F2	A, B	-
204	F1	A	-
	F2	A	-
205	F1	A, B	-
	F2	A	-
	F3	A	-
206	F1	B	-
	F2	B	-
	F3	B	-
